# Dexmedetomidine attenuates Alzheimer’s pathogenesis by targeting the ROS-mediated XIAP-MDM2-p53 signaling axis

**DOI:** 10.3389/fphar.2026.1811373

**Published:** 2026-04-17

**Authors:** Weiwei Wu, Hui Wang, Luyao Zhang, Danqing Li, Yuxiang Chen, Bolin Lin, Huiqin Jiang, Ting Meng, Zheao Zhu, Lijun He, Hui Zhang, Hu Liu, Ruijie Zhang

**Affiliations:** 1 Department of Anesthesiology,The First Affiliated Hospital of Anhui Medical University (Anhui Public Health Clinical Center), Hefei, Anhui, China; 2 College of Life Sciences, Anhui Medical University, Hefei, Anhui, China; 3 Department of Anesthesiology, The First Affiliated Hospital of Anhui Medical University, Hefei, Anhui, China; 4 Department of Anesthesiology, The Second Affiliated Hospital of Anhui Medical University, Hefei, Anhui, China

**Keywords:** dexmedetomidine, morris water maze, mouse double minute 2 homolog, poly-D-lysine, tumor protein p53

## Abstract

**Introduction:**

Alzheimer’s disease (AD) is a multifactorial neurodegenerative disorder with a complex pathological process, in which oxidative stress serves as a key pathogenic mechanism. Studies have shown that the anesthetic adjuvant dexmedetomidine (Dex) can improve postoperative cognitive function in AD patients. This study aimed to explore whether dexmedetomidine alleviates AD-associated neuronal apoptosis and cognitive impairment via reducing overproduction of ROS and regulating the XIAP signaling pathway.

**Methods:**

*In vitro* experiments were conducted using Aβ1-42-exposed SH-SY5Y cells and primary neurons, employing interventions such as the ROS scavenger NAC, yohimbine pre-treatment, and siRNA-mediated XIAP knockdown. *In vivo* cognitive deficits and brain pathology were evaluated in AD model mice using Morris water maze tests and immunofluorescence staining.

**Results:**

Experimental results demonstrated that Aβ1-42 induced apoptosis in neuronal cells, while dexmedetomidine incubation significantly reduced Aβ1-42 elicited ROS generation, activated XIAP, suppressed MDM2 and ameliorated P53 overactivation, thereby effectively preventing neuronal death. Combined administration of NAC and dexmedetomidine reversed Aβ1-42-induced XIAP inhibition, ROS accumulation, and cell apoptosis. Furthermore, both yohimbine pre-treatment and XIAP knockdown effectively abrogated the ability of Dex to reduce ROS accumulation and mitigate apoptosis. *In vivo* results indicated that dexmedetomidine improved cognitive deficits and intervened in AD pathology in the hippocampal region of AD model mice.

**Conclusion:**

This study reveals that dexmedetomidine inhibits ROS release and activates the XIAP-MDM2-p53 signaling pathway, thereby delaying apoptosis and ameliorating cognitive impairment in AD progression.

## Introduction

Alzheimer’s disease is a progressive neurodegenerative disorder, neuropathologically characterized by amyloid-β plaques and neurofibrillary tangles (Selkoe, 2001; Scheltens et al., 2016). Although numerous studies have been conducted, effective treatments for the disease are still lacking, which underscores the critical necessity of exploring new pathogenic mechanisms and discovering potential therapeutic approaches.

The pathological mechanisms underlying AD involves a complex interplay of mechanisms, including mitochondrial dysfunction, neuroinflammation, and critically, persistent oxidative stress (Swerdlow, 2018; Butterfield and Halliwell, 2019; Gao et al., 2025). Increased generation of ROS is an early and sustained event in AD, which not only directly damages neuronal macromolecules but also exacerbates the toxicity of Aβ and tau, ultimately driving neuronal apoptosis (Butterfield and Boyd-Kimball, 2018; Cheignon et al., 2018; Fanlo-Ucar et al., 2024). Cellular defense against apoptosis is orchestrated by key regulators such as the X-linked inhibitor of apoptosis protein (XIAP), an effective endogenous inhibitor of caspase activation (Deveraux and Reed, 1999; Li et al., 2001). Notably, XIAP expression is significantly downregulated in Aβ-exposed neuronal cells and in AD brains, correlating with increased neuronal vulnerability (Kugler et al., 2000; Garranzo-Asensio et al., 2018; Barber et al., 2023). Recent studies have further elucidated the intricate interplay between Tau, MDM2, and p53 in AD pathology, revealing that Tau binds to MDM2 and modulates p53 stability (Sola et al., 2023; Zhang et al., 2026). This suggests that the impairment of anti-apoptotic machinery, particularly involving XIAP and the MDM2-p53 axis, is a pivotal event in AD progression.

p53, a major tumor suppressor, plays a central role in stress-mediated apoptosis and is associated with neuronal death in AD (Nelson and Xu, 2023; Soelter et al., 2024). p53 activity is tightly regulated by MDM2, which promotes its ubiquitination and degradation (Haupt et al., 1997). Intriguingly, XIAP can act as an E3 ubiquitin ligase for MDM2, targeting it for proteasomal degradation and thereby indirectly stabilizing p53 (Sun et al., 2012; Wade et al., 2013). Our prior work demonstrated that mitochondrial ROS-induced XIAP inactivation leads to MDM2 accumulation, p53 upregulation, and apoptosis in a cadmium toxicity model (Zhao et al., 2020). However, whether this specific ROS-XIAP-MDM2-p53 signaling axis is activated and contributes to Aβ-induced neurodegeneration in AD remains unknown. Elucidating this pathway could provide a novel mechanistic link between oxidative stress and apoptotic execution in AD.

Dexmedetomidine (Dex), a clinically used α2-adrenergic receptor agonist, has demonstrated neuroprotective properties in various neurological contexts (Ma et al., 2004). Importantly, Dex is a potent antioxidant that can suppress excessive ROS generation and mitigate ROS-dependent apoptosis (Zheng et al., 2021). Its ability to ameliorate cognitive dysfunction in susceptible populations further suggests its potential relevance in AD-related pathology (Ding et al., 2021). Given its antioxidant efficacy and the hypothesized role of ROS and XIAP-MDM2-p53 axis in AD, we postulated that Dex might protect against AD by specifically targeting this pathway.

Therefore, this research intended to examine Dex’s protective effects on AD pathology and to determine whether its mechanism involves the modulation of the ROS-XIAP-MDM2-p53 signaling axis. We employed Aβ1-42-treated SH-SY5Y cells and primary neurons for *in vitro* mechanistic studies and the 5×FAD transgenic mouse model for *in vivo* validation of cognitive function and neuropathology.

## Materials and methods

### Reagents

Aβ1-42 was obtained from QYAOBIO (Shanghai, China). Dulbecco’s Modified Eagle’s Medium (DMEM) and NEUROBASAL Media were from Invitrogen (Grand Island, NY, United States). 2′7′-dichlorodihydrofluorescein diacetate (H_2_DCFDA), Dexmedetomidine, 4′,6-diamidino-2-phenylindole (DAPI), and protease inhibitor cocktail were purchased from Sigma (St Louis, MO, United States). Fetal bovine serum (FBS) was obtained from Hyclone (Logan, UT, United States). Yohimbine was purchased from MedChemExpress (Monmouth Junction, NJ, United States). CAT, NAC and poly-D-lysine (PDL)was obtained from Beyotime (Shanghai, China). The following antibodies were used: XIAP (Cell Signaling Technology, Beverly, MA, United States), Aβ1-42, MDM2, cleaved-caspase-3 and p53 (Abcam, Cambridge, MA, United States), β-tubulin, goat anti-rabbit IgG-horseradish peroxidase, goat anti-mouse IgG-HRP (Pierce, Rockford, IL, United Statesa). All remaining reagents were of analytical grade and acquired from local commercial vendors, unless noted otherwise.

### Drugs and mice

Twelve 5×FAD mice aged 20–24 weeks (mean body weight: 25 ± 2 g) were used in this study,and randomly assigned to experimental groups, with each group containing both male and female mice. Besides, WT mice were acquired from Aniphe Biolaboratory Inc. All animals were raised in a specific pathogen-free environment under standardized settings and a 12-h light-dark cycle, simultaneously keep the temperature at 25 ± 2 °C. For the *in vivo* treatment regimen, the 5×FAD mice and WT littermates received daily intraperitoneal injections of Dex at a dose of 4 μg/kg or an equal volume of saline (vehicle) for 4 consecutive weeks. To evaluate the sustained neuroprotective effects rather than acute pharmacological sedation, behavioral testing commenced 24 h after the final injection. Furthermore, to eliminate subjective bias, strict blinding procedures were implemented. The investigators who performed the Morris water maze testing, tissue processing, and subsequent histological analyses were completely blinded to the animal group allocations. All animal procedures were approved by the Ethics Committee of Anhui Medical University (Animal Ethics Approval No. 20190403). 5×FAD mice were purchased from Shanghai Model Organisms Center.

### Cell culture

Human neuroblastoma SH-SY5Y cell lines were from American Type Culture Collection (ATCC) (Manassas, VA, United States). SH-SY5Y cells were maintained in antibiotic-free DMEM enriched 10% FBS, in an incubator with controlled humidity (37 °C, 5% CO_2_). Primary neurons were harvested from the cerebral cortex of embryonic day 16–18 ICR mouse fetuses as previously reported^[26]^. After 6 days in culture, cells were plated at a density of 5 × 10^5^ cells/well in 6-well plates pre-coated with 10 μg/mL PDL for subsequent assays.

### Transient knockdown of XIAP by siRNA transfection

To establish XIAP knockdown *in vitro*, small interfering RNAs (siRNAs) specifically targeting human XIAP (si-XIAP) and a non-targeting scrambled negative control siRNA (si-NC). For experiments, SH-SY5Y cells were seeded in culture plates and allowed to reach 50%–70% confluence. Subsequently, the cells were transiently transfected with either si-XIAP or si-NC. Following a 48- to 72-h incubation, the cells were subjected to subsequent functional assays. The knockdown efficiency of endogenous XIAP was verified by Western blotting using a specific anti-XIAP antibody.

### Western blot analysis

Following the designated treatments, cells were gently rinsed with ice-cold PBS and lysed using RIPA lysis buffer. Western blot analysis was then conducted as reported earlier (Zhang et al., 2017), and the relative expression levels of target proteins were semi-quantified with NIH ImageJ software.

### Annexin-V-FITC/PI staining

SH-SY5Y cells and primary neurons were plated into 6-well plates at 5 × 10^5^ cells per well. The subsequent day, cells were first exposed to Aβ1-42 (5 μM) for 24 h or not. Subsequently, they were treated with or without Dex (10 μM) for 1 h. Subsequent to staining, the samples were washed three times by PBS and analyzed by Annexin V-FITC/PI staining to quantify the proportions of viable, early apoptotic, late apoptotic, and necrotic cells. Flow cytometric (FACS) analysis was carried out in accordance with the manufacturer’s instructions using a CytoFLEX S instrument (Beckman, United States) and an apoptosis detection kit (Beyotime, Shanghai, China).

### DAPI and TUNEL staining

SH-SY5Y cells and primary neurons were individually plated at 5 × 10^5^ cells per well in 6-well plates. After 24 h, cells were incubated with or without 5 μM Aβ1-42 for 24 h, and then treated with Dex (2.5, 5 and 10 μM) alone or in combination with NAC (5 mM) for another 1 h. Each treatment group was set up in 5 replicates. Later, cells with condensed and fragmented nuclei were stained with DAPI (4 μg/mL) following the previously described protocol (Chen et al., 2008), or after DAPI staining, using the TUNEL staining kit from Beyotime to stain (Shanghai, China). Following all staining steps, images were captured using a Leica DMi8 fluorescence microscope (Wetzlar, Germany) equipped with a digital camera.Quantitative analysis of TUNEL fluorescence intensity was performed by measuring the integral optical density with Image-Pro Plus 6.0 software.

### Intracellular ROS imaging

H_2_DCFDA was employed to visualize intracellular ROS levels. SH-SY5Y cells and primary neurons were plated at 5 × 10^5^ cells per well, each of which was furnished with a PDL-coated glass coverslip. After 24 h, cells were incubated with or without 5 μM Aβ1-42 for 24 h, and then treated with Dex (2.5, 5 and 10 μM) alone or in combination with NAC (5 mM), or yohimbine (5 μM), or a H_2_O_2_-scavenging enzyme CAT (350 U/ml), or H_2_O_2_ (0.5 mM) for another 1 h. Post-treatment, cells were incubated with H_2_DCFDA for 1 h. All stained specimens were then washed 3 times with PBS, imaged via a fluorescence microscope, and the integral optical density (IOD) of fluorescence intensity was quantitatively measured using Image-Pro Plus 6.0 software.

### Immunofluorescence and imaging

S-SY5Y cells and primary neurons were cultured at 5 × 10^5^ cells per well in 6-well or 12-well plates. After 24 h, cells were pretreated with/without Aβ1-42 (5 μM) for 24 h, and treated with/without Dex (10 μM) and/or NAC (5 mM) for 1 h. This “rescue” paradigm was designed to mimic a clinically relevant scenario where therapeutic intervention (Dex) is initiated after the pathological insult (Aβ accumulation) has already occurred, thereby assessing its potential to halt or reverse ongoing neurodegenerative processes. Next, the cells were fixed with 4% paraformaldehyde, blocked with serum containing 0.3% Triton X-100 for 1 h, and then exposed to an XIAP antibody at 4 °C for more than 24 h. After incubation, coverslips were washed 3 times with PBS for 5 min each, the cells were incubated with goat anti-rabbit IgG at room temperature for 1 h. All stained specimens were then washed 3 times with PBS, imaged via a fluorescence microscope, and the IOD of fluorescence intensity was quantitatively measured using Image-Pro Plus 6.0 software. During brain slice immunohistochemical experiments, mice are euthanized, and their brains are removed, fixed, and dehydrated for frozen sectioning; 25–30 μm cryosections were prepared, and immunohistochemical staining was conducted according to a previously reported protocol (Xu et al., 2021). Finally, fluorescence images were observed under an inverted fluorescence microscope, and IOD was quantitatively analyzed in 100–200 cells per field using Image-Pro Plus 6.0 software.

### Morris water maze (MWM)

Morris water maze test was conducted as previously reported (Xu et al., 2021). Overall, mice were trained for five consecutive days to locate a submerged escape platform (10 cm in diameter, positioned 1 cm below the water surface) in a circular water maze tank. The platform was fixed in the center of the southwest quadrant.Each mouse underwent 4 trials daily. They were released from varying entry points (north, east, southeast, and northwest) facing the pool wall, with the starting order randomized each day. Each trial lasted up to 60 s; swimming was video-tracked automatically until the mouse found and stayed on the platform for at least 2 s. Mice that failed to find the platform within the time limit were gently guided to it and allowed to remain there for 10 s.Following the visible platform trials, a probe test was performed. After removing the platform, each mouse was released from the northeast quadrant, and its swimming behavior was recorded for 30 s.

### Statistical analysis

Data are presented as the mean ± standard error (SE). Comparisons between two groups were performed using an unpaired Student’s t-test. For multiple comparisons, one-way or two-way analysis of variance (ANOVA) was performed, followed by Bonferroni post hoc tests. A p-value <0.05 was regarded as statistically significant.

## Results

### Dex attenuates Aβ1-42 induce apoptosis in neuronal cells

**FIGURE 1:**
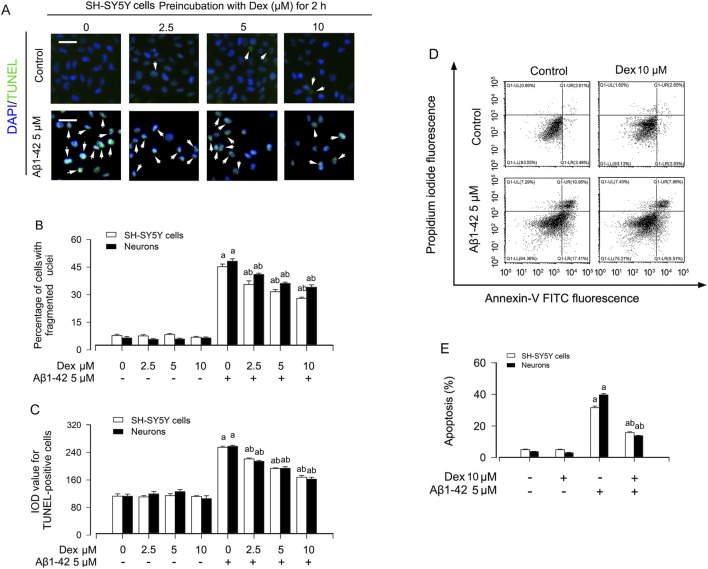
Dexmedetomidine attenuates Aβ1-42-induced apoptosis in neuronal cells. **(A)** Representative fluorescence images of SH-SY5Y cells and primary neurons stained by DAPI (blue) and TUNEL (green) after Aβ1-42 (5 μM, 24 h) exposure with or without dexmedetomidine (Dex; 0–10 µM) pretreatment. White arrows indicate cells with nuclear fragmentation or TUNEL positivity. Scale bars are included. **(B,C)** Quantification of the percentage of cells with fragmented nuclei and the integrated optical density (IOD) of TUNEL-positive signals. Data represent mean ± SE from independent experiments. **(D)** Representative Annexin V-FITC/propidium iodide (PI) flow cytometry plots under the indicated treatments, distinguishing early (Annexin V+/PI-) and late (Annexin V+/PI+) apoptotic populations. **(E)** Quantification of the percentage of apoptotic cells from flow cytometry analysis. Statistical significance was assessed using one-way ANOVA with subsequent Bonferroni post-hoc tests. Data are presented as mean ± SE (n = 5 per group). ^a^ *p* < 0.05 vs. Control; ^b^ *p* < 0.05 vs. Aβ1-42 group.

Mounting evidence supports that Dexmedetomidine can also enhance the activity of anti-apoptotic signaling cascades, attenuating neuronal damage and neurological impairments. In this study, we first examined whether Dex prevents neuronal apoptosis in AD cell models. SH-SY5Y cells and primary neurons werepre-incubated with/without Aβ1-42 (5 μM) for 24 h, exposed to Dex (2.5, 5 and 10 μM) for 1 h. As depicted in Figure 1A, imaged results revealed that Aβ1-42 markedly increased the percentage of the cells with nuclear fragmentation and condensation and the TUNEL-positive cells with fragmented DNA in SH-SY5Y cells and primary neurons. The quantitative results also support these findings (Figures 1B,C). At the same time, we found that Dex at a concentration of 10 μM inhibited Aβ1-42-induced apoptosis. So, we pretreated Aβ1-42-induced neuronal cells with Dex and detected apoptosis using flow cytometry. As shown in Figures 1D,F, Dex reversed Aβ1-42-induced apoptosis. Based on this dose-response profile, the 10 μM concentration, which provided maximal and statistically significant protection, was selected for subsequent mechanistic experiments. While this concentration is higher than clinically relevant plasma levels, it is consistent with standard cell culture practices to achieve robust and reproducible effects within the experimental timeframe and is within the range widely employed in mechanistic studies of dexmedetomidine neuroprotection (Cho et al., 2022).

### Dex plays a critical role in modulating Aβ1-42-induced ROS production, XIAP inactivation/MDM2-p53 activation, and neuronal apoptosis

**FIGURE 2:**
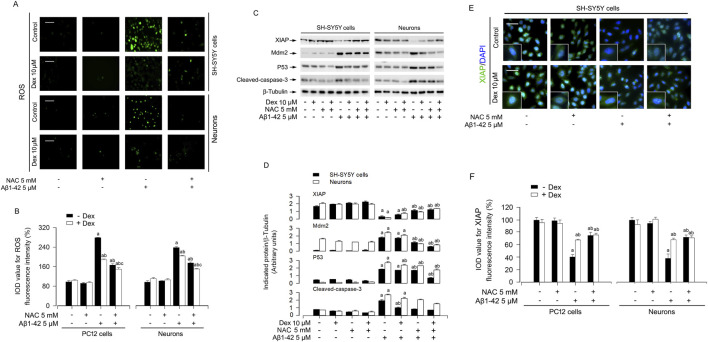
Dex suppresses Aβ1-42-triggered ROS generation and modulates the XIAP-MDM2-p53 axis. **(A)** Representative fluorescence images of intracellular ROS detected by H2DCFDA probe in SH-SY5Y cells and primary neurons following Aβ1-42 (5 μM, 24 h) stimulation with or without Dex (10 µM) and/or NAC(5 mM) pretreatment. **(B)** Quantitative analysis of ROS fluorescence intensity (IOD). **(C)** Representative immunoblots for XIAP, p53, MDM2 and cleaved-caspase-3 expression in SH-SY5Y cells and neurons. β-Tubulin was employed as an internal control. **(D)** Densitometric quantification of the indicated protein bands normalized to β-Tubulin. **(E)** Typical immunofluorescence images showing XIAP (red) localization and expression intensity with DAPI nuclear counterstaining (blue) in SH-SY5Y cells. **(F)** Quantification of XIAP fluorescence intensity (IOD) across treatment groups. Statistical significance was assessed using one-way ANOVA with subsequent Bonferroni post-hoc tests. Data are presented as mean ± SE (n = 5 per group). ^a^ *p* < 0.05 vs. Control; ^b^ *p* < 0.05 vs. Aβ1-42 group.

**FIGURE 3:**
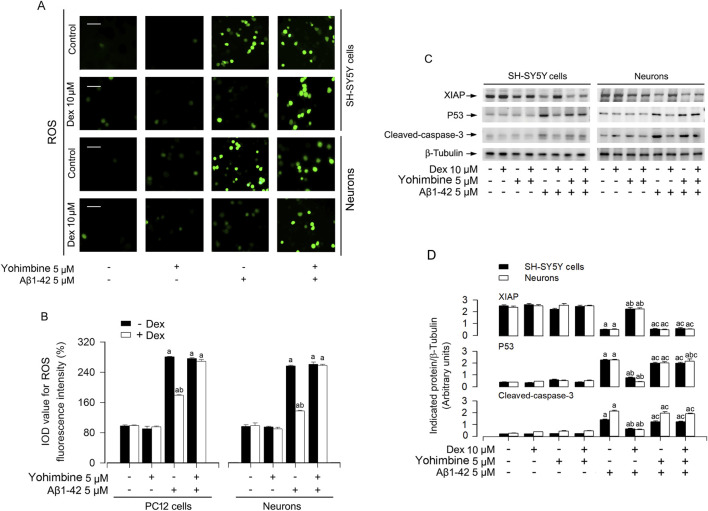
Pharmacological blockade with yohimbine abolishes the anti-oxidative and anti-apoptotic efficacy of Dexmedetomidine. **(A)** Representative fluorescence images of intracellular ROS detected by H_2_DCFDA probe in SH-SY5Y cells and primary neurons following Aβ1-42 (5 μM, 24 h) stimulation with or without Dex (10 µM) and/or yohimbine (5 µM) pretreatment. **(B)** Quantitative analysis of ROS fluorescence intensity (IOD). **(C)** Representative immunoblots for XIAP, p53 and cleaved-caspase-3 expression in SH-SY5Y cells and neurons. β-Tubulin was employed as an internal control. **(D)** Densitometric quantification of the indicated protein bands normalized to β-Tubulin. Statistical significance was assessed using one-way ANOVA with subsequent Bonferroni post-hoc tests. Data are presented as mean ± SE (n = 5 per group). ^a^ *p* < 0.05 vs. Control; ^b^ *p* < 0.05 vs. Aβ1-42 group; ^c^ *p* < 0.05, yohimbine + Aβ1-42 vs. - Dex + yohimbine + Aβ1-42 group.

**FIGURE 4:**
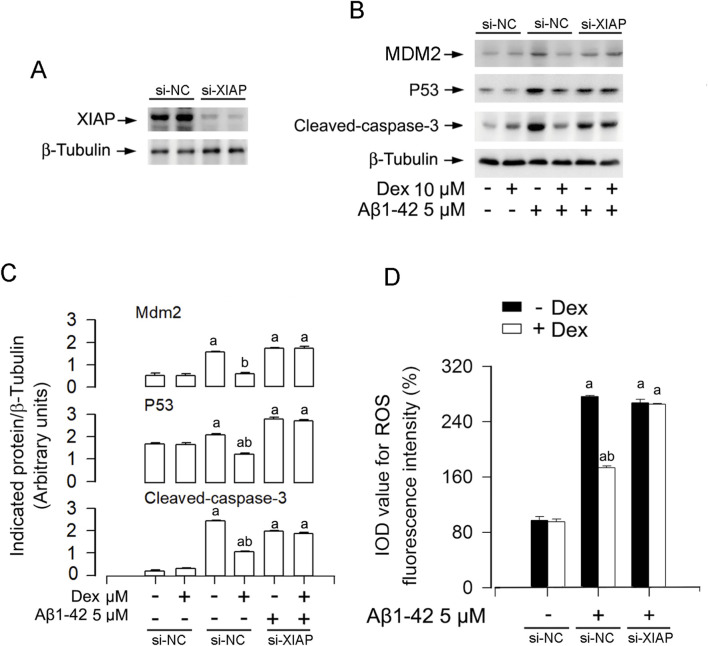
Knockdown of XIAP abolishes the anti-oxidative and anti-apoptotic effects of Dexmedetomidine. **(A)** SH-SY5Y cells were transiently transfected with either si-XIAP or si-NC. Following a 48- to 72-h incubation, the cells were subjected to Western blot analysis to detect XIAP. β-Tubulin was employed as an internal control. **(B)** Representative immunoblots for MDM2, p53 and cleaved-caspase-3 expression in cells transfected with either si-XIAP or si-NC, following Aβ1-42 (5 μM, 24 h) stimulation with or without Dex (10 µM) pretreatment. **(C)** Densitometric quantification of the indicated protein bands normalized to β-Tubulin. **(D)** Quantitative analysis of ROS fluorescence intensity (IOD). Statistical significance was assessed using one-way ANOVA with subsequent Bonferroni post-hoc tests. Data are presented as mean ± SE (n = 5 per group). ^a^ *p* < 0.05 vs. Si-NC group; ^b^ *p* < 0.05, si-NC group vs. - Dex + si-NC group.

Accumulating evidence indicates that the development of AD was accompanied by the production of excessive ROS(Rustichelli et al., 2024; Guo et al., 2025). To investigate whether ROS mediates Aβ-induced XIAP downregulation, we combined Dex (10 μM) with the ROS scavenger NAC (5 mM), hydrogen peroxide (H_2_O_2_, 0.5 mM), or a H_2_O_2_-scavenging enzyme, catalase (CAT, 350 U/ml). The results showed that Dex and NAC significantly weakened Aβ1-42 elevated ROS accumulation in neurons (Figures 2A,B). Moreover, the combination of CAT and Dex enhanced the inhibition of ROS production by Dex (Supplementary Figure S1A). As shown in Supplementary Figure S1B, Dex not only weakened the ROS production induced by Aβ1-42, but also reduced the level of H_2_O_2_.Western blot analysis showed that Aβ1-42 suppressed XIAP and upregulated MDM2 and p53, effects that were reversed by Dex and/or NAC (Figures 2C,D). SH-SY5Y cells and primary neurons were pretreated with/without Aβ1-42 (5 μM) for 24 h, and treated with/without Dex (10 μM) and/or NAC (5 mM) for 1 h, followed by analyzing the cellular protein levels of XIAP, Mdm2 and p53 using Western blotting. Results showed that with Aβ1-42 (5 μM) repressed the XIAP and activated MDM2 and p53. Simultaneously, dex reversed the expression levels of these proteins (Figures 2C,D). In addition, Our immunofluorescence staining revealed a reduction in XIAP (green) in cells exposed to 5 μM Aβ1-42, and this was prevented by Dex (Figure 2E). As shown in Figure 2F, the quantitative results also support the phenomena we observed. Collectively, Dex treatment reversed Aβ1-42-induced XIAP downregulation, ROS accumulation, and upregulation of MDM2 and p53 in neuronal cells. To determine whether the neuroprotective efficacy of Dex, a highly selective α2-adrenergic receptor agonist, is dependent on its canonical receptor pathway, AD cell models were co-treated with Dex and the specific antagonist yohimbine. As illustrated in Figures 3A,B, the Dex-mediated suppression of ROS generation in Aβ1-42 exposed cells was significantly reversed by yohimbine. Consistent with the phenotypic ROS changes, Western blot analysis (Figures 3C,D) demonstrated that yohimbine effectively negated the protective modulations of Dex at the protein level. Specifically, the Dex-induced restoration of XIAP expression and the concurrent suppression of p53 and cleaved-caspase-3 activation were completely blunted upon α2-receptor blockade. These findings indicate that the neuroprotective efficacy of Dex is fundamentally mediated through α2-adrenergic receptor activation rather than independent off-target effects. To definitively establish the central role of XIAP in Dex mediated neuroprotection, we performed targeted knockdown of XIAP using siRNA (Figure 4A). As shown in Figures 4B,C, the downstream modulation of MDM2, p53, and cleaved-caspase-3 by Dex was substantially blunted upon XIAP knockdown. Consistent with these molecular alterations, the protective effects of Dex against Aβ1-42 induced ROS generation were also significantly abolished (Figure 4D). Taken together, these data strongly indicate that Dex exerts its anti-oxidative and anti-apoptotic properties fundamentally through the XIAP-dependent signaling axis.

### Dex improves cognitive dysfunction in AD mouse model

The 5×FAD transgenic mouse, a widely utilized model in AD research, exhibits the early onset and rapidly progressive disease course (Oakley et al., 2006). These mice develop amyloid peptide deposition accompanied by neuroinflammation as early as 2 months of age, followed by neuronal decline apparent at 4 months, which worsens with age along with progressive cognitive dysfunction. To evaluate the efficacy of Dex treatment, we comprehensively assessed the cognitive function of AD mice by the morris water maze. In the first week, training was conducted with the platform visible; in the second week, training was performed with the platform hidden; and in the third week, the platform was displaced to observe whether the mice would linger and explore in the target quadrant and to record the number of platform crossings.

**FIGURE 5:**
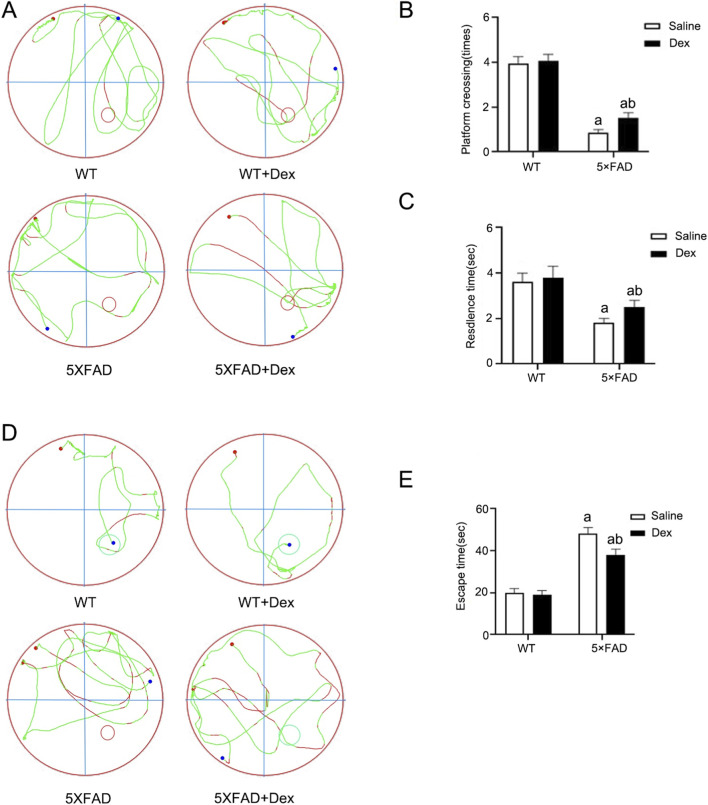
Dexmedetomidine improves spatial learning and memory in 5×FAD mice. **(A)** Representative swimming tracks in the Morris water maze probe test for WT, WT + Dex, 5×FAD, and 5×FAD + Dex groups. Red dots indicate the start point; blue dots indicate the end point. **(B)** Quantification of platform crossing times during the probe test. **(C)** Quantification of time spent in the target quadrant (residence time) during the probe test. **(D)** Representative swimming tracks during hidden platform trials. **(E)** Escape latency (escape time) measured in seconds across the indicated groups. Data are presented as mean ± SE (n = 12 per group). ^a^ *p* < 0.05 vs. WT group; ^b^ *p* < 0.05, 5×FAD groupvs - 5×FAD + Dex group.

In the spatial probe test, the 5×FAD + Dex group exhibited a significant increase in both the time spent in the target quadrant and the number of platform crossings relative to the 5×FAD group (Figures 5A–C). There was no significant difference between WT + Dex group and WT group, indicating that Dex administration does not alter the baseline cognitive performance of healthy wild-type mice. These results indicate that Dex treatment significantly ameliorates cognitive impairment in AD model mice. Then in the escape latency test, compared with the WT group, the AD model group exhibited prolonged escape latency, while Dex-treated 5×FAD mice showed shortened escape latency relative to the AD model mice (Figures 5D,E). These results indicate that Dex improved the memory and learning abilities of AD model mice.

**FIGURE 6:**
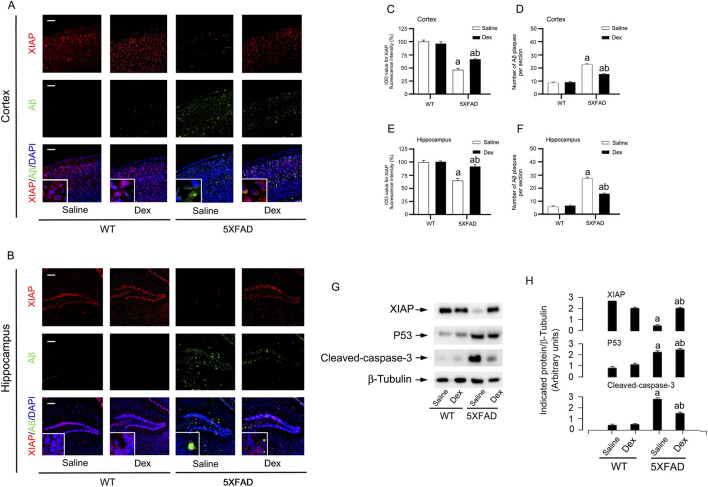
Dex reduces amyloid pathology and restores XIAP expression in the cortex and hippocampus of 5×FAD mice. **(A,B)** Representative immunofluorescence images of cortex **(A)** and hippocampus **(B)** sections showing XIAP (red), Aβ plaques (green), and merged channels with DAPI (blue) in WT and 5×FAD mice treated with Saline or Dex. Insets show higher magnification views. **(C–F)** Quantification of XIAP fluorescence intensity (IOD) and the number of Aβ plaques per section in the cortex **(C,D)** and hippocampus **(E,F)**. **(G)** Representative immunoblots of XIAP, p53, and cleaved caspase-3 with β-Tubulin as a internal control in tissue homogenates from WT and 5×FAD mice. **(H)** Quantification of band intensities relative to β-Tubulin. Data represent mean ± SE. ^a^ *p* < 0.05 vs. WT group; ^b^ *p* < 0.05, 5×FAD groupvs - 5×FAD + Dex group.

Further research revealed that immunohistochemical results indicated that Dex treatment prevented the deposition of Aβ protein plaques in the cerebral cortex of AD model mice. Additionally, Dex significantly reversed the decrease in XIAP protein expression in the AD model mice (Figures 6A,C,D). The immunofluorescence imaging and quantitative results of the hippocampus also led to the same conclusion (Figures 6B,E,F). To further confirm the changes in XIAP, the expression levels of XIAP, p53, and Cleaved-caspase-3 in WT and AD mice after dex treatment were detected by WB (Figures 6G,H). The results showed that Dex treatment could increase the level of XIAP and decrease the level of p53 and cleaved-caspase-3 in cerebral cortex and hippocampus. In summary, Dex treatment effectively alleviates neuronal apoptosis and reduces Aβ protein deposition in the hippocampal tissue of AD mice, alongside higher XIAP expression.

## Discussion

Alzheimer’s disease (AD) pathogenesis is driven by intertwined processes including Aβ aggregation, tau pathology, oxidative stress, mitochondrial dysfunction, and neuroinflammation (Huang et al., 2016; Chen et al., 2022; Adnan et al., 2025). While oxidative stress is a persistent and early event, the precise molecular cascades linking it to apoptotic neuronal loss remain incompletely elucidated. This study demonstrates that Dex confers neuroprotection in AD models by targeting a specific ROS-dependent apoptotic pathway. We show that in Aβ1-42-challenged neuronal cultures, Dex pretreatment suppresses excessive ROS generation, restores the expression of the anti-apoptotic protein XIAP, and attenuates the activation of MDM2 and p53, culminating in reduced apoptosis. Consistently, in 5×FAD transgenic mice, Dex administration upregulates hippocampal and cortical XIAP, diminishes cleaved-caspase-3 levels and Aβ plaque load, and rescues spatial learning and memory deficits. Collectively, these data provide the first evidence that Dex exerts multi-faceted neuroprotective effects in AD through modulating the “ROS-XIAP-MDM2-p53” signaling axis, highlighting a novel and targetable pathway connecting oxidative stress to neuronal apoptosis in neurodegeneration (Cenini et al., 2019).

Previous studies has shown that Dex has a protective effect on nerves (Li et al., 2024; Tao et al., 2024), and the antioxidant efficacy of Dex within the specific context of Aβ-induced AD pathology. More importantly, it moves beyond this general observation to delineate a specific downstream signaling cascade through which antioxidant effects translate into anti-apoptotic outcomes. We identify the XIAP-MDM2-p53 axis as a critical pathway mediating Dex’s neuroprotective action, thereby providing a novel mechanistic link between its established antioxidant function and the suppression of neuronal apoptosis in AD. It has been established that oxidative stress is an early and persistent event in AD, directly damaging neurons and exacerbating the toxicity of Aβ and Tau(Watt et al., 2010; Kozin, 2023). For instance, excessive ROS triggers lipid peroxidation, DNA damage, and promotes neuroinflammation and apoptosis by activating multiple signaling pathways such as JNK/p38 MAPK and NF-κB. Our study complements this understanding by clarifying that the antioxidant action of Dex serves as a key upstream event in its neuroprotection.

A key insight from this study is the central role of XIAP. While downregulation of XIAP in AD brains has been associated with increased neuronal vulnerability, recent studies have demonstrated that Dex treatment can attenuate neuronal death and cognitive decline in AD models through mechanisms involving the regulation of inflammatory and apoptotic pathways (Christie et al., 2007; Barber et al., 2023). However, the upstream regulators of XIAP in the context of AD, particularly the link to oxidative stress, remain poorly defined. This study establishes Aβ-induced ROS burst as a major upstream suppressor of XIAP and identifies Dex, via its ROS-scavenging activity, as an effective means to restore XIAP expression and function (Figures 1–4). This directly couples the antioxidant property of Dex to the preservation of a pivotal endogenous anti-apoptotic defense, offering a refined perspective on cell fate regulation in AD. Furthermore, although p53-mediated apoptosis is implicated in AD (Checler and Alves da Costa, 2014), the regulatory interplay between ROS, XIAP, MDM2, and p53 has been unclear. Our results elucidate this interplay: by clearing ROS and restoring XIAP, Dex enhances MDM2 ubiquitination and degradation, leading to decreased p53 stability and attenuation of its pro-apoptotic activity. It is important to address the regulatory interplay between MDM2 and p53 in our model. Classically, MDM2 functions as an E3 ubiquitin ligase that negatively regulates p53 by targeting it for proteasomal degradation. However, our data demonstrate a concurrent downregulation of both MDM2 and p53 following Dex treatment. This apparent paradox can be explained by the specific cellular response to severe pathological stress. Previous studies have shown that the accumulation of ROS during AD leads to neuronal death accompanied by an increase in p53 expression levels (Culmsee and Mattson, 2005; Chang et al., 2012). Because the MDM2 gene is a direct transcriptional target of p53 (Wu et al., 1993), the hyperactivation of stabilized p53 inevitably drives a robust compensatory upregulation of MDM2 expression, leading to their simultaneous accumulation in pathological states. By restoring XIAP and quenching ROS, Dex effectively extinguishes the upstream oxidative stress signal, leading to the deactivation of p53 and the subsequent transcriptional downregulation of MDM2. Crucially, our pharmacological blockade experiments utilizing yohimbine confirm that the Dex-induced modulation of the ROS-XIAP-MDM2-p53 axis is not merely a non-specific antioxidant effect, but is strictly dependent on the activation of the α2-adrenergic receptor (Figure 3).

Based on our experimental findings, we propose the following mechanistic framework. Aβ oligomers act as a key trigger, inducing a burst of oxidative stress. Dex potently suppresses this ROS burst, likely through protecting mitochondrial integrity or inhibiting membrane-bound ROS-generating enzymes like NOX (Shi et al., 2021). The resultant reduction in oxidative stress helps restore the cellular redox balance, which may stabilize XIAP protein and promote its expression, potentially via modulation of redox-sensitive post-translational modifications (Kairisalo et al., 2011; Zhou et al., 2021; Zhang et al., 2023). As an E3 ubiquitin ligase, XIAP then targets MDM2 for K48-linked polyubiquitination and proteasomal degradation. The observed decrease in MDM2 levels following Dex treatment relieves its inhibition on p53 transcriptional activity and attenuates p53 degradation, leading to reduced p53 protein stability and pro-apoptotic function. Consequently, the transcriptional activation of downstream pro-apoptotic genes is diminished. The final outcome is a marked reduction in the activation of executioner caspase-3 and in overall apoptosis, as validated by TUNEL and Annexin V/PI assays. This proposed pathway integrates our molecular, cellular, and *in vivo* observations into a coherent model explaining Dex’s neuroprotective effects in AD.

Although this study systematically elucidated the neuroprotective mechanism of Dex in AD models, we used the classic AD mouse model 5×FAD to explore whether Dex improved behavioral and pathological characterization (Figures 5, 6), but there are still some limitations. First, the research primarily relies on the Aβ pathology dominant 5×FAD model, which adequately simulates early-stage amyloid plaque deposition and associated cognitive decline in AD but exhibits relatively mild tauopathy. Therefore, the effect of Dex on abnormal tau phosphorylation, aggregation, and the resulting synaptic dysfunction and neuronal loss remains unclear. Future validation of Dex’s efficacy is warranted in models encompassing more complete AD pathology, such as the 3×Tg-AD or tau transgenic mice. Second, in this study, an equal number of male and female 5×FAD mice were used and randomly allocated to each experimental group. While our current data demonstrated the overall neuroprotective efficacy of Dex, we acknowledge that the progression of AD is highly sexually dimorphic. Due to the limited sample size for sex-stratified analysis in this cohort, potential sex-specific responses to Dex treatment were not fully explored. Future investigations specifically powered to assess sex as a biological variable are warranted to delineate whether the ROS-mediated XIAP-MDM2-p53 signaling axis operates differently between sexes in AD pathogenesis. Furthermore, while our data establish that the ROS-XIAP-MDM2-p53 pathway governs the anti-apoptotic and neuroprotective effects of Dex, this specific cascade does not directly account for the observed reduction in Aβ plaque burden.

We acknowledge that the attenuation of amyloid pathology is an intriguing but unexpected observation in the current study. The specific mechanisms driving this phenotype remain unelucidated. It is entirely speculative at this stage whether this reduction stems from a direct modulation of APP processing (such as altering β- or γ-secretase activity) or an indirect enhancement of microglial Aβ clearance. Therefore, these hypotheses warrant targeted future investigations to bridge the gap between Dex-mediated anti-apoptotic signaling and amyloid metabolism. Our *in vitro* experimental design employed a “rescue” paradigm (Aβ pre-exposure for 24 h followed by Dex for 1 h), which was intentionally chosen to model a clinically relevant scenario. But, this design does not address whether Dex can prevent the initiation of oxidative damage. Therefore, rather than interpreting this as a direct anti-amyloid effect of Dex, we view this finding as an exploratory observation that generates a testable hypothesis for future studies. Targeted investigations employing models of amyloid metabolism, such as APP processing assays or microglial depletion strategies, are warranted to determine whether this phenomenon reflects a direct pharmacological action or an indirect consequence of reduced neuronal injury. Finally, while our genetic and pharmacological data support the functional requirement of XIAP in regulating MDM2 and p53, direct biochemical evidence—such as co-IP assays to confirm XIAP-MDM2 interaction—remains to be established in the AD context.

In conclusion, this study identifies the ROS-XIAP-MDM2-p53 signaling axis as a critical mechanistic pathway through which dexmedetomidine alleviates Aβ-induced neuronal apoptosis and cognitive impairment in models of Alzheimer’s disease. Ultimately, our work enriches the current understanding of AD pathology and encourages further exploration of dexmedetomidine as a neuroprotective strategy.

## Data Availability

The raw data supporting the conclusions of this article will be made available by the authors, without undue reservation.

## Supplementary material

The Supplementary Material for this article can be found online at: https://www.frontiersin.org/articles/10.3389/fphar.2026.1811373/full#supplementary-material

SUPPLEMENTARY FIGURE S1 Dexmedetomidine suppresses Aβ1-42- and H2O2-triggered oxidative stress, an effect enhanced by catalase co-treatment. (C) (A and B) Quantitative analysis of ROS fluorescence intensity (IOD) from fluorescence images of intracellular ROS detected by H2DCFDA probe in SH-SY5Y cells and primary neurons following Aμ1-42 (5 βM, 24 h) stimulation with or without Dex (10 µM) and/or CAT (350 U/ml) and/or H2O2(0.5 mM) pretreatment. Statistical significance was assessed using one-way ANOVAwith subsequent Bonferroni post-hoc tests. Data are presented as mean ± SE (n=5 per group). ap < 0.05 vs. Control; bp < 0.05 vs.Aβ1-42 group; cp < 0.05, CAT +Aβ1-42 vs- Dex + CAT+Aβ1-42 group, or H2O2 vs- Dex + H2O2 group.

## References

[B1] Adnan M. Siddiqui A. J. Bardakci F. Surti M. Badraoui R. Patel M. (2025). Neuroprotective potential of quercetin in alzheimer's disease: targeting oxidative stress, mitochondrial dysfunction, and amyloid-beta aggregation. Front. Pharmacol. 16, 1593264. 10.3389/fphar.2025.1593264 40567363 PMC12187643

[B2] Barber K. Mendonca P. Soliman K. F. A. (2023). The neuroprotective effects and therapeutic potential of the chalcone cardamonin for alzheimer's disease. Brain Sci. 13 (1), 145. 10.3390/brainsci13010145 36672126 PMC9856590

[B3] Butterfield D. A. Boyd-Kimball D. (2018). Oxidative stress, amyloid-beta peptide, and altered key molecular pathways in the pathogenesis and progression of alzheimer's disease. J. Alzheimers Dis. 62 (3), 1345–1367. 10.3233/JAD-170543 29562527 PMC5870019

[B4] Butterfield D. A. Halliwell B. (2019). Oxidative stress, dysfunctional glucose metabolism and alzheimer disease. Nat. Rev. Neurosci. 20 (3), 148–160. 10.1038/s41583-019-0132-6 30737462 PMC9382875

[B5] Cenini G. Lloret A. Cascella R. (2019). Oxidative stress in neurodegenerative diseases: from a mitochondrial point of view. Oxid. Med. Cell. Longev. 2019, 2105607. 10.1155/2019/2105607 31210837 PMC6532273

[B6] Chang J. R. Ghafouri M. Mukerjee R. Bagashev A. Chabrashvili T. Sawaya B. E. (2012). Role of p53 in neurodegenerative diseases. Neurodegener. Dis. 9 (2), 68–80. 10.1159/000329999 22042001 PMC3304514

[B7] Checler F. Alves da Costa C. (2014). p53 in neurodegenerative diseases and brain cancers. Pharmacol. Ther. 142 (1), 99–113. 10.1016/j.pharmthera.2013.11.009 24287312

[B8] Cheignon C. Tomas M. Bonnefont-Rousselot D. Faller P. Hureau C. Collin F. (2018). Oxidative stress and the amyloid beta peptide in alzheimer's disease. Redox Biol. 14, 450–464. 10.1016/j.redox.2017.10.014 29080524 PMC5680523

[B9] Chen L. Liu L. Luo Y. Huang S. (2008). MAPK and mTOR pathways are involved in cadmium-induced neuronal apoptosis. J. Neurochem. 105 (1), 251–261. 10.1111/j.1471-4159.2007.05133.x 18021293

[B10] Chen X. Chen D. Chen P. Chen A. Deng J. Wei J. (2022). Dexmedetomidine attenuates apoptosis and neurological deficits by modulating neuronal NADPH oxidase 2-Derived oxidative stress in neonates following hypoxic brain injury. Antioxidants (Basel) 11 (11), 2199. 10.3390/antiox11112199 36358571 PMC9686745

[B11] Cho I. Koo B. N. Kim S. Y. Park S. Kim E. J. Kam E. H. (2022). Neuroprotective effect of dexmedetomidine against postoperative cognitive decline *via* NLRP3 inflammasome signaling pathway. Int. J. Mol. Sci. 23 (15), 8806. 10.3390/ijms23158806 35955939 PMC9369249

[B12] Christie L. A. Su J. H. Tu C. H. Dick M. C. Zhou J. Cotman C. W. (2007). Differential regulation of inhibitors of apoptosis proteins in alzheimer's disease brains. Neurobiol. Dis. 26 (1), 165–173. 10.1016/j.nbd.2006.12.017 17292615 PMC2198925

[B13] Culmsee C. Mattson M. P. (2005). p53 in neuronal apoptosis. Biochem. Biophys. Res. Commun. 331 (3), 761–777. 10.1016/j.bbrc.2005.03.149 15865932

[B14] Deveraux Q. L. Reed J. C. (1999). IAP family proteins-suppressors of apoptosis. Genes. Dev. 13 (3), 239–252. 10.1101/gad.13.3.239 9990849

[B15] Ding X. D. Cao Y. Y. Li L. Zhao G. Y. (2021). Dexmedetomidine reduces the lidocaine-induced neurotoxicity by inhibiting inflammasome activation and reducing pyroptosis in rats. Biol. Pharm. Bull. 44 (7), 902–909. 10.1248/bpb.b20-00482 34193687

[B16] Fanlo-Ucar H. Picon-Pages P. Herrera-Fernandez V. Ill-Raga G. Munoz F. J. (2024). The dual role of amyloid beta-peptide in oxidative stress and inflammation: unveiling their connections in alzheimer's disease etiopathology. Antioxidants (Basel) 13 (10), 1208. 10.3390/antiox13101208 39456461 PMC11505517

[B17] Gao M. Dai M. T. Gong G. H. (2025). Dysfunctional glucose metabolism triggers oxidative stress to induce kidney injury in diabetes. World J. Diabetes 16 (4), 102554. 10.4239/wjd.v16.i4.102554 40236851 PMC11947919

[B18] Garranzo-Asensio M. San Segundo-Acosta P. Martinez-Useros J. Montero-Calle A. Fernandez-Acenero M. J. Haggmark-Manberg A. (2018). Identification of prefrontal cortex protein alterations in alzheimer's disease. Oncotarget 9 (13), 10847–10867. 10.18632/oncotarget.24303 29541381 PMC5834268

[B19] Guo X. Zhang B. Chen Y. Jia Z. Yuan X. Zhang L. (2025). Multifunctional mesoporous nanoselenium delivery of metformin breaks the vicious cycle of neuroinflammation and ROS, promotes microglia regulation and alleviates alzheimer's disease. Colloids Surf. B Biointerfaces 245, 114300. 10.1016/j.colsurfb.2024.114300 39447310

[B20] Haupt Y. Maya R. Kazaz A. Oren M. (1997). Mdm2 promotes the rapid degradation of p53. Nature 387 (6630), 296–299. 10.1038/387296a0 9153395

[B21] Huang W. J. Zhang X. Chen W. W. (2016). Role of oxidative stress in alzheimer's disease. Biomed. Rep. 4 (5), 519–522. 10.3892/br.2016.630 27123241 PMC4840676

[B22] Kairisalo M. Bonomo A. Hyrskyluoto A. Mudo G. Belluardo N. Korhonen L. (2011). Resveratrol reduces oxidative stress and cell death and increases mitochondrial antioxidants and XIAP in PC6.3-cells. Neurosci. Lett. 488 (3), 263–266. 10.1016/j.neulet.2010.11.042 21094207

[B23] Kozin S. A. (2023). Role of interaction between zinc and amyloid beta in pathogenesis of alzheimer's disease. Biochem. (Mosc) 88 (Suppl. 1), S75–S87. 10.1134/S0006297923140055 37069115

[B24] Kugler S. Straten G. Kreppel F. Isenmann S. Liston P. Bahr M. (2000). The X-linked inhibitor of apoptosis (XIAP) prevents cell death in axotomized CNS neurons *in vivo* . Cell. Death Differ. 7 (9), 815–824. 10.1038/sj.cdd.4400712 11042676

[B25] Li J. Feng Q. Kim J. M. Schneiderman D. Liston P. Li M. (2001). Human ovarian cancer and cisplatin resistance: possible role of inhibitor of apoptosis proteins. Endocrinology 142 (1), 370–380. 10.1210/endo.142.1.7897 11145600

[B26] Li R. Zhang Y. Zhu Q. Wu Y. Song W. (2024). The role of anesthesia in peri-operative neurocognitive disorders: molecular mechanisms and preventive strategies. Fundam. Res. 4 (4), 797–805. 10.1016/j.fmre.2023.02.007 39161414 PMC11331737

[B27] Ma D. Hossain M. Rajakumaraswamy N. Arshad M. Sanders R. D. Franks N. P. (2004). Dexmedetomidine produces its neuroprotective effect *via* the alpha 2A-adrenoceptor subtype. Eur. J. Pharmacol. 502 (1-2), 87–97. 10.1016/j.ejphar.2004.08.044 15464093

[B28] Nelson T. J. Xu Y. (2023). Sting and p53 DNA repair pathways are compromised in alzheimer's disease. Sci. Rep. 13 (1), 8304. 10.1038/s41598-023-35533-6 37221295 PMC10206146

[B29] Oakley H. Cole S. L. Logan S. Maus E. Shao P. Craft J. (2006). Intraneuronal beta-amyloid aggregates, neurodegeneration, and neuron loss in transgenic mice with five familial alzheimer's disease mutations: potential factors in amyloid plaque formation. J. Neurosci. 26 (40), 10129–10140. 10.1523/JNEUROSCI.1202-06.2006 17021169 PMC6674618

[B30] Rustichelli S. Lanni C. Zara M. Guidetti G. F. Torti M. Canobbio I. (2024). Curcumin modulates platelet activation and ROS production induced by amyloid peptides: new perspectives in attenuating prothrombotic risk in Alzheimer's Disease patients. Nutrients 16 (24), 4419. 10.3390/nu16244419 39771040 PMC11678805

[B31] Scheltens P. Blennow K. Breteler M. M. de Strooper B. Frisoni G. B. Salloway S. (2016). Alzheimer's disease. Lancet 388 (10043), 505–517. 10.1016/S0140-6736(15)01124-1 26921134

[B32] Selkoe D. J. (2001). Alzheimer's disease: genes, proteins, and therapy. Physiol. Rev. 81 (2), 741–766. 10.1152/physrev.2001.81.2.741 11274343

[B33] Shi J. Yu T. Song K. Du S. He S. Hu X. (2021). Dexmedetomidine ameliorates endotoxin-induced acute lung injury *in vivo* and *in vitro* by preserving mitochondrial dynamic equilibrium through the HIF-1a/HO-1 signaling pathway. Redox Biol. 41, 101954. 10.1016/j.redox.2021.101954 33774474 PMC8027777

[B34] Soelter T. M. Howton T. C. Clark A. D. Oza V. H. Lasseigne B. N. (2024). Altered glia-neuron communication in alzheimer's disease affects WNT, p53, and NFkB signaling determined by snRNA-seq. Cell. Commun. Signal 22 (1), 317. 10.1186/s12964-024-01686-8 38849813 PMC11157763

[B35] Sola M. Rendon-Angel A. Rojo Martinez V. Sgrignani J. Magrin C. Piovesana E. (2023). Tau protein binds to the P53 E3 ubiquitin ligase MDM2. Sci. Rep. 13 (1), 10208. 10.1038/s41598-023-37046-8 37353565 PMC10290082

[B36] Sun X. X. Challagundla K. B. Dai M. S. (2012). Positive regulation of p53 stability and activity by the deubiquitinating enzyme otubain 1. EMBO J. 31 (3), 576–592. 10.1038/emboj.2011.434 22124327 PMC3273389

[B37] Swerdlow R. H. (2018). Mitochondria and mitochondrial cascades in alzheimer's disease. J. Alzheimers Dis. 62 (3), 1403–1416. 10.3233/JAD-170585 29036828 PMC5869994

[B38] Tao Z. Li P. Zhao X. (2024). Progress on the mechanisms and neuroprotective benefits of dexmedetomidine in brain diseases. Brain Behav. 14 (11), e70116. 10.1002/brb3.70116 39482839 PMC11527817

[B39] Wade M. Li Y. C. Wahl G. M. (2013). MDM2, MDMX and p53 in oncogenesis and cancer therapy. Nat. Rev. Cancer 13 (2), 83–96. 10.1038/nrc3430 23303139 PMC4161369

[B40] Watt N. T. Whitehouse I. J. Hooper N. M. (2010). The role of zinc in alzheimer's disease. Int. J. Alzheimers Dis. 2011, 971021. 10.4061/2011/971021 21197404 PMC3010690

[B41] Wu X. Bayle J. H. Olson D. Levine A. J. (1993). The p53-mdm-2 autoregulatory feedback loop. Genes. Dev. 7 (7A), 1126–1132. 10.1101/gad.7.7a.1126 8319905

[B42] Xu C. Wu J. Wu Y. Ren Z. Yao Y. Chen G. (2021). TNF-alpha-dependent neuronal necroptosis regulated in alzheimer's disease by coordination of RIPK1-p62 complex with autophagic UVRAG. Theranostics 11 (19), 9452–9469. 10.7150/thno.62376 34646380 PMC8490500

[B43] Zhang R. Zhang N. Zhang H. Liu C. Dong X. Wang X. (2017). Celastrol prevents cadmium-induced neuronal cell death by blocking reactive oxygen species-mediated mammalian target of rapamycin pathway. Br. J. Pharmacol. 174 (1), 82–100. 10.1111/bph.13655 27764525 PMC5341486

[B44] Zhang H. Jin B. Liu L. Li H. Zheng X. Li M. (2023). Glutathione might attenuate arsenic-induced liver injury by modulating the Foxa2-XIAP axis to reduce oxidative stress and mitochondrial apoptosis. Biol. Trace Elem. Res. 201 (11), 5201–5212. 10.1007/s12011-023-03577-4 36689145

[B45] Zhang Z. Zhang M. Cao Z. Zhao H. Li X. Luo P. (2026). Fibrillarin: bridging ribosome biogenesis and apoptosis in cellular stress and disease. Apoptosis 31 (1), 11. 10.1007/s10495-025-02220-y 41518572

[B46] Zhao R. Yu Q. Hou L. Dong X. Zhang H. Chen X. (2020). Cadmium induces mitochondrial ROS inactivation of XIAP pathway leading to apoptosis in neuronal cells. Int. J. Biochem. Cell. Biol. 121, 105715. 10.1016/j.biocel.2020.105715 32035180 PMC7045337

[B47] Zheng T. Zheng C. Gao F. Huang F. Hu B. Zheng X. (2021). Dexmedetomidine suppresses bupivacaine-induced parthanatos in human SH-SY5Y cells *via* the miR-7-5p/PARP1 axis-mediated ROS. Naunyn Schmiedeb. Arch. Pharmacol. 394 (4), 783–796. 10.1007/s00210-020-01971-6 32989562

[B48] Zhou Y. Long M. Y. Chen Z. Q. Huang J. W. Qin Z. B. Li L. (2021). Downregulation of miR-181a-5p alleviates oxidative stress and inflammation in coronary microembolization-induced myocardial damage by directly targeting XIAP. J. Geriatr. Cardiol. 18 (6), 426–439. 10.11909/j.issn.1671-5411.2021.06.007 34220972 PMC8220381

