# Supplementary figures and images for: Dexmedetomidine attenuates Alzheimer’s pathogenesis by targeting the ROS-mediated XIAP-MDM2-p53 signaling axis
Source: Front Pharmacol. 2026 Apr 17;17:1811373. doi: 10.3389/fphar.2026.1811373 (PMC13132828; doi:10.3389/fphar.2026.1811373)

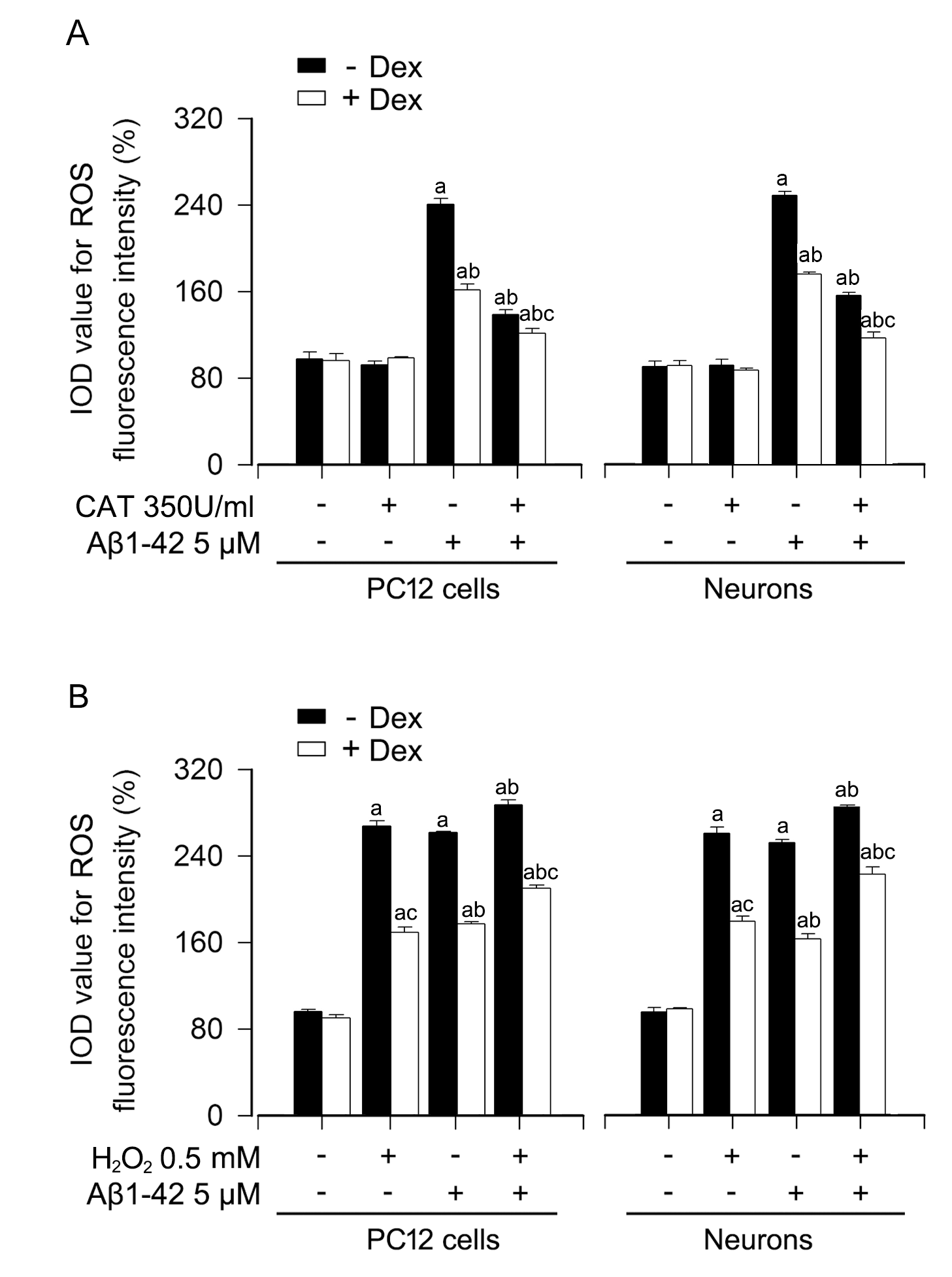

Supplement: Supplementary file 1 [file Image1.tif]
